# Mitigating Future Avian Malaria Threats to Hawaiian Forest Birds from Climate Change

**DOI:** 10.1371/journal.pone.0168880

**Published:** 2017-01-06

**Authors:** Wei Liao, Carter T. Atkinson, Dennis A. LaPointe, Michael D. Samuel

**Affiliations:** 1 Department of Forestry and Wildlife Ecology, University of Wisconsin-Madison, Madison, Wisconsin, United States of America; 2 U. S. Geological Survey, Pacific Island Ecosystems Research Center, Hawai’i National Park, Hawai’i, United States of America; 3 U. S. Geological Survey, Wisconsin Cooperative Wildlife Research Unit, University of Wisconsin, Madison, Wisconsin, United States of America; Johns Hopkins University Bloomberg School of Public Health, UNITED STATES

## Abstract

Avian malaria, transmitted by *Culex quinquefasciatus* mosquitoes in the Hawaiian Islands, has been a primary contributor to population range limitations, declines, and extinctions for many endemic Hawaiian honeycreepers. Avian malaria is strongly influenced by climate; therefore, predicted future changes are expected to expand transmission into higher elevations and intensify and lengthen existing transmission periods at lower elevations, leading to further population declines and potential extinction of highly susceptible honeycreepers in mid- and high-elevation forests. Based on future climate changes and resulting malaria risk, we evaluated the viability of alternative conservation strategies to preserve endemic Hawaiian birds at mid and high elevations through the 21^st^ century. We linked an epidemiological model with three alternative climatic projections from the Coupled Model Intercomparison Project to predict future malaria risk and bird population dynamics for the coming century. Based on climate change predictions, proposed strategies included mosquito population suppression using modified males, release of genetically modified refractory mosquitoes, competition from other introduced mosquitoes that are not competent vectors, evolved malaria-tolerance in native honeycreepers, feral pig control to reduce mosquito larval habitats, and predator control to improve bird demographics. Transmission rates of malaria are predicted to be higher than currently observed and are likely to have larger impacts in high-elevation forests where current low rates of transmission create a refuge for highly-susceptible birds. As a result, several current and proposed conservation strategies will be insufficient to maintain existing forest bird populations. We concluded that mitigating malaria transmission at high elevations should be a primary conservation goal. Conservation strategies that maintain highly susceptible species like Iiwi (*Drepanis coccinea*) will likely benefit other threatened and endangered Hawai’i species, especially in high-elevation forests. Our results showed that mosquito control strategies offer potential long-term benefits to high elevation Hawaiian honeycreepers. However, combined strategies will likely be needed to preserve endemic birds at mid elevations. Given the delay required to research, develop, evaluate, and improve several of these currently untested conservation strategies we suggest that planning should begin expeditiously.

## Introduction

Endemic Hawaiian honeycreepers (*Passeriformes Drepanididae*) were once abundant in forests throughout Hawai’i [1]. Unfortunately today, most of these species are extinct, have severely reduced populations, or are restricted to high elevation forests. Hawaiian forest birds are considered one of the most threatened group of forest-dependent birds in the world [2]. There are multiple causes for their declines including habitat destruction, and the introduction of predators and avian competitors [3]. However, introduced avian malaria (*Plasmodium relictum*) transmitted by the southern house mosquitoes (*Culex quinquefasciatus*) is considered to be a key factor limiting present distribution and abundance of the remaining Hawaiian native birds [4–8]. Because malaria dynamics are strongly influenced by ambient temperature and precipitation patterns [9–12], predicted future climate changes (i.e., increasing temperature and altered rainfall) are expected to increase the occurrence, distribution, and intensity of avian malaria transmission to Hawaiian forest birds [8,13,14].

A comprehensive epidemiological model of the Hawaiian forest bird-malaria system illustrates that transmission varies across altitudes on Hawai’i Island; from high year-round transmission in warm lowland forests, to seasonal episodic transmission at mid elevation, and infrequent summer infections at cool high elevations [13]. Consequently, predicted climate change will substantially alter the geographic and elevational distribution of malaria pathogens by facilitating transmission in high-elevation forests, currently with low disease transmission, and increasing transmission at lower elevations [12,15,16]. In the Hawaiian Islands, warming has already been observed at higher elevations [17], where cool seasonal temperatures previously inhibited *Plasmodium* development in mosquitoes [18]. Malaria prevalence may have recently increased in endemic birds at high elevations in Hawai’i [19–21], which constitute the only remaining refugia for native species that are highly susceptible to malaria, supporting the concern that native Hawaiian forest birds may suffer further population declines and/or species extinction due to climate-induced increase in disease transmission [8,11,13,14,22–25].

To evaluate the altitudinal and temporal impacts of future climate change on avian malaria transmission in Hawaiian forest birds, we previously [14] linked three climate projections representing warm and dry (RCP 4.5), warm and wet (A1B), and hot and dry (RCP 8.5) futures, with our epidemiological model [13] to predict malaria prevalence, mosquito abundance, and bird dynamics until the end of the 21^st^ century. Fortini et al. [25] evaluated climate associated habitat changes to estimate habitat suitability for Hawaiian birds during the next century. Both studies indicated that future climate change will likely cause an increase in malaria transmission [14], reduction in suitable habitat [25], and decrease in the abundance of the remaining Hawaiian honeycreepers. In high-elevation forests, malaria transmission is predicted to become more frequent and intense during the second half of this century, resembling seasonal epidemics currently found in mid-elevation forests [14]. As a result, even for the most optimistic climatic projection, malaria sensitive birds will be increasingly exposed to malaria infection, and likely suffer severe population declines and/or extinctions. In mid-elevation forests, seasonal transmission becomes longer and more intense for all three climatic projections, and may lead to mid-elevation extinction for disease susceptible Iiwi populations, which have already been decimated by malaria. In contrast to mid and high elevations, lowland forests have high malaria transmission because this is a highly favorable environment for mosquitoes and malaria transmission [14,26–27]. Therefore, predicted future climate changes will have only limited additional impacts on malaria transmission at low elevation. Recent studies have demonstrated that only the Hawai’i Amakihi (*Chlorodrepanis virens*) has been able to evolve malaria tolerance and re-populate low-elevation forests in the face of high malaria transmission [21,26,28–30]; providing limited optimism that evolution of malaria tolerance may also allow other native Hawaiian birds to avoid extinction.

Given the severe consequences predicted for increased avian malaria transmission to native birds throughout the 21^st^ century, it is crucial to develop effective strategies to protect Hawaiian forest birds that also consider future climate change scenarios [8,14,25]. Several potential mitigation strategies have been proposed to accomplish the conservation goal of reducing avian malaria impacts, including: improving bird demographics to counter malaria [31], reducing vector abundance [32], and decreasing malaria transmission from mosquitoes to birds [32]. We used our epidemiological model and alternative future climate scenarios [13,14] to evaluate disease management strategies with a goal of maintaining current Hawaiian forest bird populations until 2100. We considered two alternatives to improve bird demographics: evolution of malaria-tolerance (reduced malaria mortality) in native bird species and predator removal to increase bird survival and reproduction. We also evaluated potential reduction in mosquito abundance by: controlling feral pigs which create larval mosquito habitat; introducing vector-incompetent mosquitoes which compete with *Culex* larvae for resources; and releasing either sterile male mosquitoes or male mosquitoes infected with incompatible *Wolbachia* strains to reduce mosquito reproduction and abundance. Finally, we considered genetically modified refractory mosquitoes that block malaria parasite development and therefore decrease disease transmission from mosquitoes to birds. We evaluated the potential effectiveness of these mitigation strategies based on population growth, the ratio of model predicted bird abundance at 2100 (future) compared to 2010 (current). Our goal was to identify potential mitigation strategies that result in stable forest bird populations in high-elevation forests or increasing bird populations in mid-elevation forests.

## Methods

### The simulation model

We evaluated the performance of different avian malaria conservation strategies on native Hawaiian forest bird populations and malaria transmission under 3 alternative future climate change projections for the 21^st^ century. We used an ordinary differential equation (ODE) epidemiological model [13,14] to estimate the daily changes in susceptible, infected, and recovered (SIR) birds, and susceptible, exposed (latent), and infectious (SEI) mosquitoes. The epidemiological model (and our evaluation) includes the vector–*C*. *quinquefasciatus*, and three native Hawaiian honeycreepers –Hawai’i Amakihi, Apapane (*Himatione sanguine*), and Iiwi (*Drepanis coccinea*). The model also includes one introduced bird—Japanese White-eye (*Zosterops japonicus*), a species that is resistant to avian malaria infection. Three climatic projections from the Coupled Model Intercomparison Project (CMIP) including phase 3 A1B, and phase 5 Representative Concentration Pathways (RCP) 4.5 and RCP8.5 scenarios for the 21^st^ century were used to drive the epidemiological model [14]. Simulations used a 24-year burn-in run with historical weather patterns, estimated malaria free bird densities, and 100 infectious adult mosquitoes per km^2^ to generate initial state values. These initial values were inputs for a 24-year baseline simulation using daily weather data from 1980–2003 (including inter-annual and inter-decadal climate variations). We predicted the future daily temperature and rainfall through linear interpolation of the projected temperature and rainfall changes between 2004 and 2100 for each of the three alternative climate projections (see details in [14]). We summarized the daily results into annual and 10-year intervals to interpret the long-term trends for bird and mosquito densities, prevalence of infected vectors and hosts, and risk of malaria infection. Simulations were performed using the ode45 solver in Matlab version R2011b (The MathWorks, Inc., Natick, MA, USA). Details of the bird-mosquito-malaria model and climate scenarios including model equations, parameter definitions and values, and climate trends are described in [13] and [14].

Our evaluation assumes implementation of conservation strategies starting in 2020 in mid-elevation, and 2030 in high-elevation forests based on predicted delays in malaria risk associated with these elevations [14]. We used the population growth rate (PGR in eq 1 below) for bird abundance to evaluate the performance of each malaria mitigation strategy. Successful conservation strategies had a PGR ≥ 1, which allows current bird populations to persist or grow. We found PGR to provide a reasonable metric for representing long-term population changes for Hawaiian honeycreepers with the exception of Iiwi populations at mid-elevation. Currently Iiwi have very low abundance in mid-elevation forests because of avian malaria; therefore, PGR values near 1 primarily indicated the population continued to exist, but was not recovering. Instead PGR >> 10 for mid-elevation Iiwi indicated this threatened population of birds was benefiting.

$$Population\;Growth\;Rate\;\left(PGR\right)\;=\;\frac{Nbird\;@\;2100}{Nbird\;@\;2010}$$ (1)

### Single mitigation strategies

#### Improvement in host demographics

Population growth rate and population persistence are positively correlated: larger population growth rates are associated with longer population persistence time and smaller risk of extinction [33]. Population growth is determined by population demographics including survival rate, sex ratio, mortality, migration, reproduction, carrying capacity, and other factors. We considered two approaches designed to increase population growth rate and persistence: 1) reduction of disease-associated mortality through evolution of malaria-tolerance and 2) improvements in adult survival and reproductive success through removal of predators.

**Malaria tolerance.** We modeled potential evolution of malaria-tolerance from theoretical models of the evolution of disease tolerance for simple Mendelian inheritance [34]. We accounted for evolution of tolerance by using separate subpopulations of malaria-tolerant and malaria-susceptible birds for each Hawaiian species. Subpopulations had identical demographics, but differed in malaria mortality. We assumed there is no fitness cost for development of malaria tolerance. For potential evolution of malaria tolerance we assumed a small number of tolerant birds were present in 2004 because our simulations showed that disease risk (selection pressure) at mid-elevation is currently large enough for successful development of malaria-tolerant native birds. In our simulations these subpopulations were allowed to grow and/or decline independently based on elevation-specific malaria transmission rates (selection pressure) and their simulated degree of malaria-tolerance. Malaria fatality rates for susceptible Amakihi (68%), Apapane (47%), and Iiwi (93%) were estimated from capture-recapture data for wild birds across high and mid elevations [26]. For malaria-tolerant Amakihi, we used a malaria fatality rate of 2.5% based on estimates for low-elevation Amakihi [26]. Without evidence for malaria tolerance in Apapane or Iiwi, we simulated three different levels of reduction (25%, 50%, and 75%) in malaria mortality.

To determine the evolutionary potential for development of a malaria-tolerant population of birds we conducted a pilot simulation based on malaria-tolerant Amakihi in low-elevation forests. We found that small initial subpopulations of malaria-tolerant birds consisting of 1%, 2% or 5% of the total population became dominant within less than 50 years, providing a reasonable representation of the observed recovery of the Amakihi at low elevation [35]. Therefore, we use a similar mix (1%, 5%, and 10%) for the initial population of malaria tolerant native birds at high and mid elevation.

**Predator removal.** Summarized nesting success from 36 studies of 19 Hawaiian forest birds showed predator removal had limited ability to improve nest success for Iiwi (2–10%) and Amakihi (10–15%), but a larger impact on Apapane (10–30%) [36]. Despite the rather equivocal data on predator control for Hawaiian birds, previous models evaluated the potential population effects of rodent control by modeling 25%, 50%, and 75% reductions in adult mortality and 20%, 35%, and 50% reductions in nest predation [31]. To estimate the potential effect of predator control on native birds, we simulated optimistic effects of predator removal by increasing bird fecundity by 5%, 10%, 25% or 50%, and simultaneously decreasing adult natural mortality by 25% or 50%.

#### Reduction of vector abundance

Previous studies indicate that reduction in vector abundance is a commonly used strategy to reduce transmission of vector-borne diseases [37–39]. For mosquitoes in the Hawaiian Islands, we considered three management approaches to reduce mosquito abundance and malaria transmission: 1) controlling feral pigs to reduce larval habitat for mosquitoes [18]; 2) releasing *Aedes japonicus* mosquitoes to compete with *Culex* mosquitoes for larvae habitat; and 3) releasing sterile male mosquitoes or male mosquitoes infected with incompatible *Wolbachia* strains that mate with wild females and thus reduce mosquito recruitment.

**Feral pig control.** Larval *Culex quinquefasciatus* use a wide variety of aquatic habitats, but Hawaiian rainforests with porous volcanic substrates and poorly developed stream drainages limit the availability of standing water habitat [18,40,41]. Feral pigs, which are managed as a game species in many parts of the state, feed on tree fern trunks creating cavities that become ideal larval mosquito habitats when they fill with leaf litter and rainwater [8]. At mid and high elevation on the southeastern slopes of Mauna Loa and Kilauea Volcanoes on Hawai’i Island, where tree ferns are common, larval carrying capacity (K_L_) is comprised of natural tree cavities and tree fern cavities created by feral pigs. Removal of feral pigs from these rain forests can decrease the abundance of larval habitat, leading to a decrease in vector abundance [8,32]. We simulated this mitigation strategy by calculating a reduced K_L_ based on larval habitats that are not created by feral pigs in mid elevation (see [13] and [42] for specific values) and used the same value for high-elevation.

**Competition with *Aedes japonicus japonicas*.** Biological control using larval predators or competitors is another potential management strategy for vector-borne diseases [8,32]. In Hawai’i, frogs, mosquito fish (Gambusia sp.), predatory mosquitoes (*Toxorhynchites sp*.), copepods, and damselfly larvae have failed to control mosquitoes and some of these introduced species had adverse impacts on native biodiversity [32,43]. As a result, we only considered an approach using *Ae*.*j*. *japonicus* mosquitoes that are potential larval competitors of *Culex*, but are not vectors of *Plasmodium relictum* (LaPointe, unpublished data). Originally from Asia, this invasive mosquito has been found in Europe [44] and dozens of U.S. states including Hawai’i [45]. It is sympatric with several *Culex* species in the continental U.S. [42], and may contribute to population declines in co-occurring *Culex* spp. [46–48]. However, there is conflicting evidence [49–52] whether *Ae*.*j*. *japonicus* is an important larval competitor with *Culex* species. Despite current uncertainty about the effectiveness of *Ae*.*j*. *japonicus* as a larval competitor with *Culex*, we simulated this strategy with 5% and 10% reductions in elevation-specific larval carrying capacity (K_L_) of *Culex* to mimic successful establishment of *Ae*.*j*. *japonicus*.

**Sterile and incompatible male mosquitoes.** The sterile male technique (SMT) has been historically used to control agricultural pests and vectors of human or livestock disease [53]. SMT is based on releasing overwhelming numbers of sterile males that are produced through irradiation, chemical treatment, or genetic modification. They compete with wild males to mate with wild females. The mating between wild females and sterile males produce few, if any, viable offspring, thus reducing the mosquito population in the next generation. Repeated applications of this strategy are aimed at substantially reducing or eradicating local vector populations. Releases of irradiated and chemically treated male mosquitoes have been used successfully at small scales [53]. However, a more promising technology is based on genetic enhancement of SMT based on insertion of dominant lethal genes (RIDL, Oxitec Ltd^®^) into the genome. Lethal genes are only expressed in the zygotes, rather than in the father, which greatly improves the ability of sterile males to compete with wild males for mates. To date the RIDL system was primarily developed for *Aedes aegypti* to control dengue fever (and subsequently Chikungunya and Zika virus). While theoretically adaptable to the avian malaria system, this strategy requires further development, research, field evaluation, and validation for *Culex* species prior to implementation.

Incompatible insect techniques based on *Wolbachia pipientis*, an obligate intracellular bacterium found in reproductive tissues of mosquitoes have been used successfully to control populations of *Culex quinquefasciatus* [54]. This approach is based on bidirectional cytoplasmic incompatibility (CI), the abortive embryonic development occurs when males infected with one strain of the bacterium are crossed with uninfected females or females infected with an incompatible strain. This can substantially decrease the number of viable eggs produced by female mosquitoes [54].

Although these two approaches are based on different mechanisms (SMT depending on lethal genes, irradiation, and chemical sterilization; CI depending on natural incompatibilities between *Culex* mosquitoes infected with different strains of *Wolbachia*), both are designed to decrease the number of viable eggs and limit mosquito recruitment. Therefore, we modeled these two approaches by assuming a proportion (P) of wild females mate with sterile males or males infected with incompatible *Wolbachia* strains, such that matings produce a smaller proportion (1- egg mortality) of viable eggs, which can develop to normal larvae. The remaining (1-P) females mate with normal males, and produce normal viable eggs. These two approaches include four parameters: the annual/seasonal time period when male mosquitoes are released, the density of male mosquitoes released (S per km^2^), the mating competitiveness between the sterile (S) or incompatible males and normal males (C), and the egg mortality achieved by the mating. The mating proportion, P, is determined by the mating competitiveness, the density/abundance of normal (N) and sterile or incompatible males, by the formula: $P=\frac{C*S}{N+C*S}$.

Studies using genetically modified *Aedes* mosquitoes and crosses of *Culex* mosquitoes infected with incompatible *Wolbachia* strains demonstrated high egg mortality in affected females (>90%), but a wide range of mating competitiveness (0.1–1) [54–60]. Therefore we simulated three scenarios for mating competitiveness 0.5, 0.8, and 0.9; and three levels of egg mortality 90%, 95%, and 99% in females that mate with modified males. Mosquitoes are more abundant during the dry season (fall) than in the wet season [13,14,18]; thus, we conducted simulations by releasing the male mosquitoes beginning in April, and continuing the release for 3, 6, 9, and 12 months. As mosquito abundance varies substantially between high and mid elevations, we started with a release number (S) of 10 times the predicted wild mosquito density (N). Using a recursive approach, we varied the number of release months and mosquitoes released until we were able to identify critical model parameter values that achieved our conservation goal (PGR ≥ 1).

#### Malaria transmission reduction with refractory transgenic mosquitoes

Refractory transgenic mosquitoes provide an emerging alternative to traditional vector control based on source reduction, insecticides, or biocontrol. Molecular tools are used to identify and transplant refractory gene(s) (conferring inability to transmit disease) inside the vector to interfere with pathogen transmission to susceptible hosts [61]. As a result, the proportion of mosquitoes that are infectious to birds is reduced, leading to reductions in malaria transmission to birds.

Considering the many uncertainties related to fitness cost of the refractory mosquitoes, development of gene drive systems, and the rate of refractory gene(s) fixation, we used a simple approach to simulate refractory mosquitoes in our model. For each day (time step), we assumed a fixed percentage of infectious mosquitoes were refractory (denoted by Ref%) in the population. Lacking a field evaluation of feeding rates of refractory mosquitoes, we assumed that susceptible birds are bitten in proportion to the relative abundance of refractory and wild infectious mosquitoes. So Ref% birds are bitten by refractory mosquitoes and these susceptible birds will not become infected with avian malaria (disease transmission rate is 0). The remaining (100%—Ref%) susceptible birds are bitten by wild mosquitoes capable of transmitting malaria [13]. We evaluated five potential levels of refractory mosquitoes (Ref% = 50%, 80%, 90%, 95%, and 100%) for both mid and high elevations.

### Combined mitigation strategies

The long history of attempted control of vector-borne diseases suggests that single solutions will not be adequate to address avian malaria transmission in Hawaii and that integrated approaches will be necessary [32,42]. Because a limited number of the single mitigation strategies are successful (see results), especially under hotter and/or wetter climatic projections (RCP8.5 and/or A1B), we also evaluated several combined mitigation strategies. We focused on two groups of combined strategies. First, feral pig removal plus either predator removal or the release of sterile males/males infected with incompatible *Wolbachia* strains. Second, evolution of malaria tolerance plus either refractory mosquitoes, feral pig control, release of sterile males/males infected with incompatible *Wolbachia* strains, or predator removal. Because it was impractical to evaluate the entire model parameter space for several of these combined approaches, we identified combined strategies with parameters that were successful in maintaining bird abundance by 2100 (PGR ≥ 1). We evaluated the combined strategies under RCP8.5, the most severe climate prediction for future malaria transmission, in high- and mid-elevation forests.

## Results

Malaria-tolerant Amakihi are the primary native honeycreeper able to thrive in lowland forests with high transmission of avian malaria [26,28–29]. Although Apapane are also found in lowland forests their abundance is limited. As a result, we did not evaluate the potential viability of conservation strategies in these low-elevation areas. For both mid and high elevations, our evaluations showed that Apapane populations were relatively stable (predicted population declines < 20%) for even the worst climatic climate projection [14]. Therefore, we focused our detailed evaluations on Amakihi and Iiwi at high and mid elevations. Of these two species, Iiwi are highly sensitive to avian malaria and provide a likely surrogate for other threatened and endangered birds that currently only reside at high elevation where avian malaria infection is minimal. Japanese White-eyes, an introduced bird, were included in our epidemiological model as a potential malaria reservoir, but we did not evaluate their population changes because they are not affected by avian malaria [13].

### Evolution of malaria tolerance

Our simulations evaluating evolution of malaria tolerance showed that population growth between 2010 and 2100 (PGR) depended on elevation-specific malaria transmission rates (selection pressure), climate change projections, species, magnitude of reduction of malaria-induced mortality, and to a lesser extent the initial frequency of tolerant birds in the population (S1 and S2 Tables). High selection pressure (high malaria transmission) tended to favor malaria tolerant birds, which increased in abundance to become the dominant portion of the population. At mid elevation, climatic projections that increased malaria transmission favor the evolution of malaria tolerant birds, which lead to an increase in bird abundance (S1 and S2 Tables). However, at high elevation, selection pressure was lower and only gradually increased during the 21^st^ century. Malaria sensitive species, like Iiwi, declined because the susceptible subpopulation dropped substantially and the tolerant subpopulation had only a small increase (PGR < 0.95 in S1 Table). As a result, evolution of malaria tolerance was more likely to be successful at mid elevation or for climate patterns that favor higher malaria transmission at high elevation (greater improvement in PGR, S1 and S2 Tables).

Population benefits derived by evolving malaria tolerance depended on the species susceptibility and the amount of reduction (tolerance) in malaria mortality. Tolerant low-elevation Amakihi had a substantial reduction in malaria mortality compared to Amakihi at higher elevations [26]. This reduced level of mortality favored the tolerant subpopulation, which became dominant (S3 Table). However, for highly susceptible Iiwi, with no evidence of malaria tolerance, future abundance depended on the magnitude of evolved reduction in malaria mortality. Unless Iiwi can evolve tolerance to malaria infection that reduces mortality by at least 50–75% in areas with high selection pressure (mid-elevation or RCP8.5/A1B in high elevation, S1 Table) they are unlikely to maintain current populations (S3 Table).

### Predator removal

Our simulations showed that Amakihi populations can benefit (PGR > 1) from relatively small demographic improvements that might be achievable with predator reduction programs (S4 Table). Even under the worst climate projections (RCP8.5) Amakihi populations improved in mid- and high-elevation forests with these relatively minor demographic improvements. In contrast, Iiwi showed continued population decline (PGR < 1) even with unrealistically large demographic improvements from predator removal (50% reduction in natural mortality and 50% increase in fecundity). Under the most optimistic climatic projection (RCP4.5) and these unrealistic demographic improvements, high-elevation Iiwi populations remained, but at reduced abundance (maximum PGR = 0.86). For more likely climatic projections (RCP8.5) high-elevation Iiwi populations declined by > 50%, even with substantial demographic improvement. Mid-elevation Iiwi, which are currently uncommon, continued to decline under most climate projections and were likely to vanish from mid-elevation forests.

### Feral pig control and *Aedes japonicus japonicus* competition

The goals of feral pig control or establishment of *Ae*. *j*. *japonicus* were to reduce the abundance of *Culex* mosquito larvae and thus the number of adult mosquitoes. Pig control reduced the quantity of larval habitat while *Ae*.*j*. *japonicus* reduced survival of *Culex* larvae through larval competition. Our simulations for pig control showed that population growth rate (PGR) varies by elevation, future climatic projections, and bird species (S5 Table). At mid elevation, climate is already favorable for mosquitoes and there is sufficient natural larval habitat, so all three climate scenarios predicted high mosquito populations with substantial future impacts on Amakihi (PGR < 0.15), and likely extinction of Iiwi (PGR < 0.05). Where natural cavities are relatively abundant, future declines in honeycreeper populations were likely even in actively managed areas such as Volcano National Park where pigs have already been eliminated. In high-elevation forests, which are climatically less favorable for mosquitoes, results from pig control differed for the three climatic projections. If the future climate is moderate (RCP4.5), pig control was effective in maintaining populations of Amakihi and Iiwi. However, if the future is more favorable for mosquitoes (A1B or RCP8.5) pig control could be beneficial to Amakihi, but it would not reverse the predicted decline in Iiwi (PGR < 0.35). Although pig control did not meet our conservation goal (PGR ≥ 1), simulations show that this strategy could postpone predicted high elevation bird population declines by two to four decades (S1 and S2 Figs).

Few studies provided strong evidence of larval competition between *Culex* and *Ae*.*j*. *japonicus* mosquitoes; therefore, we assumed only a small reduction (5–10%) in *Culex* larvae carrying capacity from establishment of *Ae*.*j*. *japonicus*. We found that the future climatic projections completely overwhelmed the potential benefits of this strategy. In high-elevation forests, our simulations showed only trivial improvement in PGR for Amakihi and Iiwi for the moderate climatic projection (RCP4.5) and almost no improvement in PGR when climate was favorable for mosquitoes (S6 Table). Not surprisingly, this strategy had no benefit at mid elevation, where the climate was more favorable for mosquito recruitment and disease transmission. An additional problem with the introduction of *Ae*.*j*. *japonicus* is that this species is a vector of West Nile Virus as well as several other encephalitis viruses [52], which could have a disastrous impact on native Hawaiian birds.

### Sterile or incompatible male mosquitoes

Use of sterile or incompatible male mosquitoes to reduce the number of the viable eggs and decrease abundance of adult mosquitoes required modification of several model parameters (see methods) each with many potential levels, resulting in hundreds of potential outcomes. To evaluate the general patterns we reported simulations that succeeded in maintaining future bird abundance (PGR ≥ 1) and compared those with similar parameters that were not successful. These results are intended to provide a guideline for mosquito release criteria to successfully maintain future bird populations.

**Fig 1:**
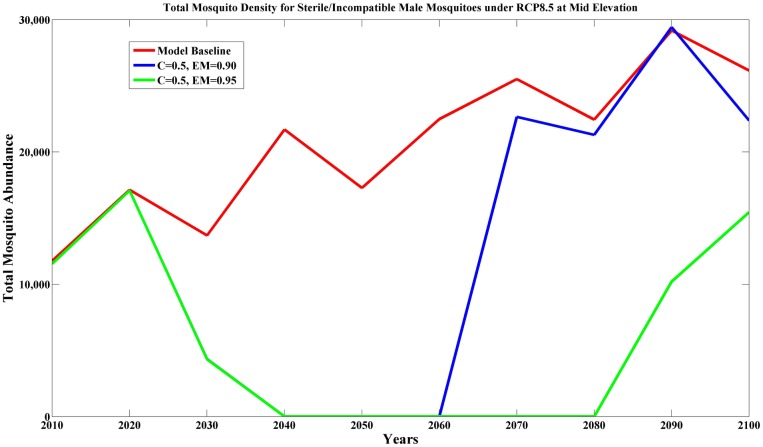
Predicted total mosquito density (per km^2^) for sterile/incompatible male mosquitoes under RCP8.5 at mid elevation. Model baseline (red line) means no release of modified (sterile or incompatible) mosquitoes, other lines show continuous release of 500 modified male mosquitoes per km^2^ for 9 months with mate competition coefficient (C = 0.5) between modified and wild male mosquito and different magnitudes of egg mortality (EM, blue line for EM = 0.90, and green line for EM = 0.95).

**Fig 2:**
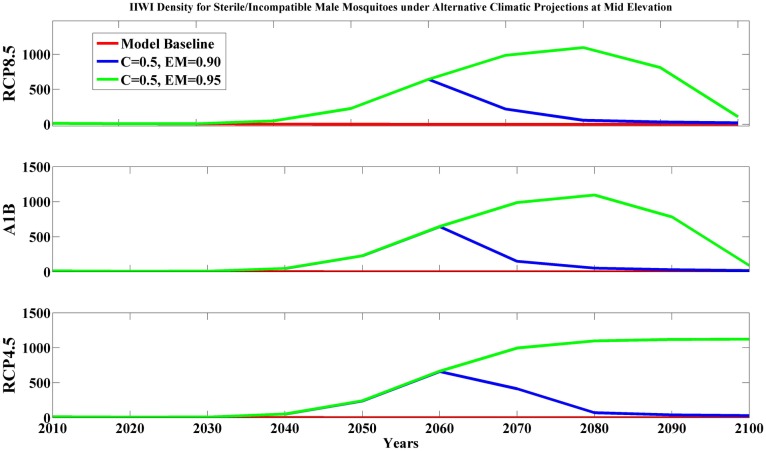
Predicted Iiwi density (per km^2^) for sterile/incompatible male mosquitoes under RCP8.5, A1B, and RCP4.5 climate projections at mid elevation. Model baseline (red line) means no release of modified (sterile or incompatible) mosquitoes, other lines show continuous release of 500 modified male mosquitoes per km^2^ for 9 months with mate competition coefficient (C = 0.5) between modified and wild male mosquito and different magnitudes of egg mortality (EM, blue line for EM = 0.90, and green line for EM = 0.95).

Our results indicated that releasing sterile/incompatible male mosquitoes can be successful at both mid elevation and high elevation (PGR > 7, S7 Table). This result was surprising given the large vector population in mid-elevation forests. However, we found that continually releasing about 500 males/km^2^ for a nine-month period resulted in dramatic reductions in mosquito abundance after 10–20 years (Fig 1). With this substantial reduction in mosquito abundance, Amakihi and Iiwi dramatically increased (PGR > 50, Fig 2). This trend persisted until later in the century when environmental conditions became more favorable for the vector and releasing 500 sterile/incompatible male mosquitoes per km^2^ no longer suppressed the wild mosquito populations. As a result of the favorable climate after 2060, mosquito abundance and malaria transmission increased causing bird populations to decline to levels closer to current abundance. However, simulations indicated the possibility that disease-sensitive species such as Iiwi could re-colonize the mid-elevation habitats if vector abundance and malaria transmission were substantially reduced.

In high-elevation forests, success of the sterile/incompatible male strategy varied with future climatic projections, bird species, and competition between released sterile/incompatible males and wild males (S7 Table). For the most optimistic climatic projection (RCP4.5), Amakihi and Iiwi had sustainable populations (PGR ≥ 1) if there was moderate mating competition (Competition = 0.5, egg mortality = 90%) between released and wild mosquitoes. If the climate was more favorable for mosquitoes (RCP8.5/A1B) or birds were more susceptible to avian malaria (Iiwi), sustaining birds until 2100 required that released mosquitoes are more competitive with wild mosquitoes. Alternatively, maintaining bird populations could also be achieved with higher egg mortality, a longer period of releasing mosquitoes, or releasing a larger number of mosquitoes (S7 Table).

#### Refractory mosquitoes

**Fig 3:**
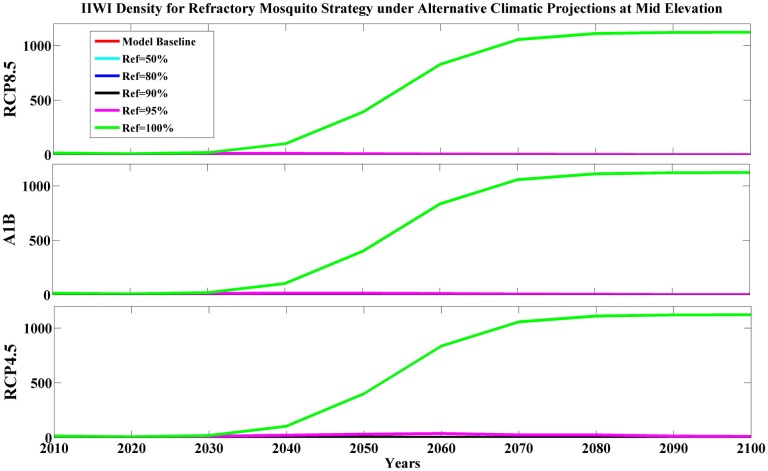
Predicted Iiwi density (per km^2^) for refractory mosquito strategy under alternative climatic projections at mid elevation. Model base (red line) means no refractory mosquitoes in the population, others imply a fixed percentage refractory mosquitoes in the population (Ref% = 50%—cyan, 80%—blue, 90%—black, 95%—magnolia and 100%—green, respectively) in alternative climatic projections (RCP8.5, A1B, and RCP4.5) of 21^st^ century.

**Fig 4:**
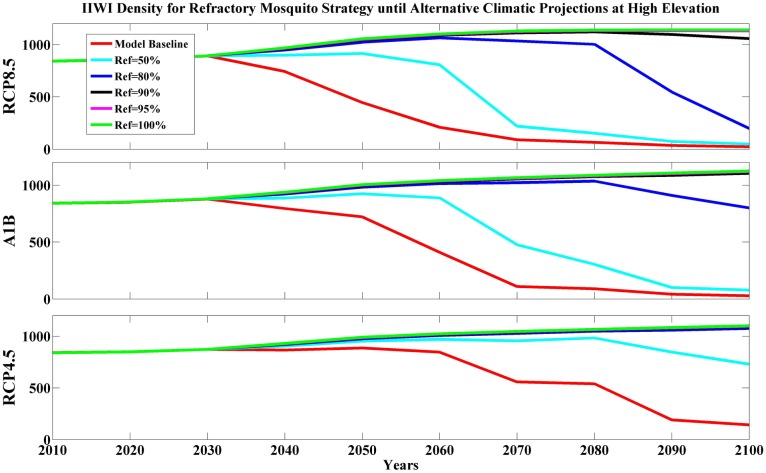
Predicted Iiwi density (per km^2^) for refractory mosquito strategy under alternative climatic projections at high elevation. Model base (red line) means no refractory mosquitoes in the population, others imply a fixed percentage refractory mosquitoes in the population (Ref% = 50%—cyan,80%—blue, 90%—black, 95%—magnolia and 100%—green, respectively) in alternative climatic projections (RCP8.5, A1B, and RCP4.5) of 21^st^ century.

Due to insufficient information on the potential application of refractory mosquitoes for controlling malaria, we simulated the infectious mosquito population where we assumed a fixed percentage (50–100%) of the mosquito population was refractory and therefore unable to transmit avian malaria to birds. Simulation results showed marginal benefits from applying this strategy at mid elevation for Iiwi and Amakihi unless wild mosquitoes were nearly eliminated (Fig 3, S8 Table). Our results indicated this strategy reduced the density of infectious mosquitoes; however, environmental conditions in mid-elevation forests are favorable enough that the remaining wild mosquito population maintained substantial malaria transmission. In high-elevation forests, the benefits of this strategy differed among climatic projections and avian species. This action was most successful for the moderate climatic projection (RCP4.5). High-elevation Amakihi benefited (PGR ≥ 1) from the strategy with a lower frequency of refractory mosquitoes (Ref% = 50% for RCP4.5, Ref% = 80% for RCP8.5, and A1B, S8 Table). In contrast, maintaining at least 90% refractory mosquitoes in the population was necessary for maintaining malaria-susceptible species in severe climates (RCP8.5 or A1B). Although maintaining a lower proportion (80%) of refractory mosquitoes did not achieve a stable population, this level delayed honeycreeper declines for several decades (Fig 4).

### Combined mitigation strategies

To evaluate potential integrated strategies and address the limited success in maintaining future bird populations using most single mitigation strategies, we also evaluated a number of combined strategies for the RCP8.5 climate projection. We simulated feral pig control in combination with predator removal or release of sterile or incompatible male mosquitoes for RCP8.5. The combined strategies of feral pig control and predator removal showed a high degree of success for Amakihi populations for both mid and high elevation and all levels of predator removal (Table 1). In contrast, this combined strategy was not successful in maintaining Iiwi populations at either elevation unless predator removal achieved unlikely demographic improvements. The combination of feral pig control with sterile or incompatible male mosquitoes predicted the most successful outcomes with substantial increases in Amakihi in both mid- and high-elevation forests under most parameter combinations (Table 2). Implementing this combined strategy also benefited disease-sensitive species, like Iiwi, at high elevation and increased their abundance at mid elevation (PGR > 1).

**Table 1:** The population growth rate (PGR) for Iiwi and Amakihi for feral pig control and predator removal in RCP8.5.

Species	Elevation	Pig Control	Pig Control Only	Predator Removal		
				50% * μ_N_ 110% * F	50% * μ_N_ 125% * F	50% * μ_N_ 150% * F
Iiwi	High	No Pig Control	0.03	0.3	0.4	0.5
		Pig Control	0.2	0.5	0.6	0.8
	Mid	No Pig Control	0.01	0.3	0.5	0.9
		Pig Control	0.01	0.5	0.7	**1.6**
Amakihi	High	No Pig Control	0.2	**2.3**	**2.7**	**3.2**
		Pig Control	0.8	**2.7**	**3.0**	**3.5**
	Mid	No Pig Control	0.1	**6.4**	**10**	**16**
		Pig Control	0.1	**7.5**	**12**	**17**

μ_N_, adult bird natural mortality (50%);

F, fecundity (increase to 110%, 125%, and 150% * F).

**Table 2:** The population growth rate (PGR) for Iiwi and Amakihi for feral pig control and release of sterile/incompatible male mosquitoes in RCP8.5.

Species	Elevation	Pig Control	Single Strategy	Modified Male Mosquito				
				M = 9 S = 500 C = 0.5 EM = 0.90	M = 9 S = 500 C = 0.5 EM = 0.95	M = 9 S = 750 C = 0.5 EM = 0.90	M = 9 S = 750 C = 0.5 EM = 0.95	M = 12 S = 750 C = 0.5 EM = 0.90
Iiwi	High	No Pig Control	0.03	0.1	0.3	0.3	0.5	0.3
		Pig Control	0.2	0.7	**1.2**	**1.1**	**1.3**	**1.3**
	Mid	No Pig Control	0.01	**1.5**	**7.6**	**1.6**	**1.6**	**79**
		Pig Control	0.01	**3.9**	**11**	**4.1**	**4.1**	**79**
Amakihi	High	No Pig Control	0.2	0.8	**1.2**	**1.1**	**1.6**	**1.2**
		Pig Control	0.8	**1.9**	**2.6**	**2.5**	**2.8**	**2.8**
	Mid	No Pig Control	0.1	**1.3**	**4.6**	**1.4**	**1.4**	**16**
		Pig Control	0.1	**2.8**	**5.6**	**2.8**	**2.8**	**16**

M (months),the number of months needed release sterile or incompatible male mosquitoes; S, the number of sterile/ incompatible male mosquitoes released into field per km^2^; C (competition), the mating competition coefficient between the sterile or incompatible and wild male mosquito; EM, the egg mortality achieved by mating between a wild female and a sterile /incompatible male.

We also simulated evolution of malaria tolerance combined with feral pig control (Table 3), refractory mosquitoes (Table 4), sterile or incompatible male mosquitoes (Tables 5 and 6), or predator removal (Table 7) for the RCP8.5 climate projection. We found additive results when malaria-tolerance was combined with predator removal. These strategies were complementary because tolerance reduced malaria mortality and predator removal reduced natural mortality and increased fecundity, thus, both improved host demographics. As a result, population levels consistently increased (PGR > 1) for both Iiwi and Amakihi at mid and high elevation (Table 7). In contrast, the success of malaria tolerance combined with feral pig control differed by elevation. At high elevation feral pig control reduced selection pressure to develop malaria-tolerance and therefore the abundance of both Iiwi and Amakihi (Table 3) also decreased. However, in mid-elevation forests, with higher selection pressure, the addition of feral pig control was either neutral or slightly positive for long-term Iiwi and Amakihi populations; malaria infection remained sufficient to favor malaria-tolerance in mid-elevation populations. The results for combining malaria tolerance with refractory mosquitoes were more complex. In high elevation where the selection pressure was generally low, a refractory mosquito percentage less than the threshold (Ref% < 90%) reduced selection pressure and prevented tolerant birds (especially Iiwi) from becoming the dominant subpopulation, so bird density declines. However, once a successful refractory mosquito threshold was achieved avian malaria was not a threat for native birds because only a small percent of mosquitoes transmitted disease to susceptible birds. Incorporating refractory mosquitoes with potential malaria tolerance at mid elevation provided only a slight advantage for development of tolerance among Hawaiian native birds (Table 4).

**Table 3:** The population growth rate (PGR) for Iiwi and Amakihi for malaria tolerance and feral pig control in RCP8.5.

Species	Elevation	Malaria Tolerance		Tolerance Only	Pig Control
		Initial Tolerant Bird Percentage	Disease Mortality (μ_D_)		
Iiwi	High	0%	93%	0.03	0.2
		1%	47%	0.4	0.2
			23%	**1.1**	0.5
		5%	47%	0.7	0.5
			23%	**1.2**	0.9
	Mid	0%	93%	0.01	0.01
		1%	47%	**4.1**	**4.2**
			23%	**63**	**64**
		5%	47%	**15**	**15**
			23%	**68**	**68**
Amakihi	High	0%	68%	0.2	0.8
		1%	2.5%	**2.1**	**1.1**
		5%	2.5%	**2.7**	**1.8**
	Mid	0%	68%	0.1	0.1
		1%	2.5%	**15**	**15**
		5%	2.5%	**15**	**15**

μ_D_, the malaria-induced mortality from Samuel et al. 2015 [26] of 93% for non-tolerant Iiwi, and 68% for non-tolerant Amakihi.

**Table 4:** The population growth rate (PGR) for Iiwi and Amakihi for malaria tolerance and refractory mosquitoes for RCP8.5.

Species	Elevation	Malaria Tolerance		Tolerance Only	Refractory Mosquito			
		Initial Tolerant Bird Percentage	Disease Mortality (μ_D_)		Ref = 80%	Ref = 90%	Ref = 95%	Ref = 100%
Iiwi	High	0%	93%	0.03	0.2	**1.3**	**1.3**	**1.4**
		1%	47%	0.3	0.3	**1.3**	**1.3**	**1.4**
			23%	**1.1**	0.3	**1.3**	**1.3**	**1.4**
		5%	47%	0.7	0.4	**1.2**	**1.3**	**1.4**
			23%	**1.2**	0.5	**1.2**	**1.3**	**1.4**
	Mid	0%	93%	0.01	0.01	0.02	0.06	**79**
		1%	47%	**4.1**	**5.4**	**8.0**	**16**	**79**
			23%	**63**	**64**	**65**	**67**	**79**
		5%	47%	**15**	**19**	**24**	**36**	**79**
			23%	**68**	**68**	**68**	**69**	**79**
Amakihi	High	0%	68%	0.3	**1.1**	**2.8**	**2.9**	**3.0**
		1%	2.5%	**2.1**	**1.2**	**2.8**	**2.9**	**3.0**
		5%	2.5%	**2.7**	**1.5**	**2.8**	**2.9**	**3.0**
	Mid	0%	68%	0.1	0.1	0.1	0.3	**16**
		1%	2.5%	**15**	**15**	**15**	**15**	**16**
		5%	2.5%	**15**	**15**	**15**	**15**	**16**

μ_D_, the malaria-induced mortality from Samuel et al. 2015 [26] of 93% for non-tolerant Iiwi, and 68% for non-tolerant Amakihi; Ref%, the infectious refractory female mosquito percentage.

**Table 5:** The population growth rate (PGR) for Iiwi and Amakihi high-elevation forests for malaria tolerance and sterile or incompatible male mosquitoes for RCP8.5.

Species	Elevation	Malaria Tolerance		Tolerance Only	Male Mosquitoes Release 750 S/km^2^ for 12 months			
		Initial Tolerant Bird Percentage	Disease Mortality (μ_D_)		C = 0.5 EM = 0.90	C = 0.8 EM = 0.90	C = 0.5 EM = 0.99	C = 0.8 EM = 0.95
Iiwi	High	0%	93%	0.03	0.3	0.8	**1.0**	**1.0**
		1%	47%	0.4	0.6	0.9	0.9	**1.2**
			23%	**1.1**	0.5	0.8	0.9	**1.1**
		5%	47%	0.7	0.4	0.9	**1.1**	**1.0**
			23%	**1.2**	0.7	0.8	**1.1**	**1.1**
Amakihi	High	0%	68%	0.2	**1.2**	**2.0**	**2.3**	**2.4**
		1%	2.5%	**2.1**	**1.8**	**2.2**	**2.2**	**2.6**
		5%	2.5%	**2.7**	**1.6**	**2.2**	**2.5**	**2.4**

μ_D_, the malaria-induced mortality from Samuel et al. 2015 [26] of 93% for non-tolerant Iiwi, and 68% for non-tolerant Amakihi; S, sterile/incompatible mosquitoes; C (competition), the mating competition coefficient between the sterile or incompatible and wild male mosquito; EM, the egg mortality achieved by mating between a wild female and a sterile/incompatible male

**Table 6:** The population growth rate (PGR) for Iiwi and Amakihi mid-elevation forests for malaria tolerance and sterile or incompatible male mosquitoes for RCP8.5.

Species	Elevation	Malaria Tolerance		Tolerance Only	Male Mosquitoes Release 500 S/km^2^ for 9 months	
		Initial Tolerant Bird Percentage	Disease Mortality (μ_D_)		C = 0.5 EM = 0.90	C = 0.5 EM = 0.95
Iiwi	Mid	0%	93%	0.01	**1.5**	**7.6**
		1%	47%	**4.1**	**19**	**12**
			23%	**63**	**63**	**31**
		5%	47%	**15**	**37**	**24**
			23%	**68**	**67**	**52**
Amakihi	Mid	0%	68%	0.1	**1.3**	**4.6**
		1%	2.5%	**15**	**12**	**7.0**
		5%	2.5%	**15**	**15**	**11**

μ_D_, the malaria-induced mortality from Samuel et al. 2015 [26] of 93% for non-tolerant Iiwi, and 68% for non-tolerant Amakihi; S, sterile/incompatible mosquitoes; C (competition), the mating competition coefficient between the sterile or incompatible and wild male mosquito; EM, the egg mortality achieved by mating between a wild female and a sterile/incompatible male

**Table 7:** The population growth rate (PGR) for Iiwi and Amakihi for malaria tolerance and predator removal for RCP8.5.

Species	Elevation	Malaria Tolerance		Tolerance Only	Predator Removal		
		Initial Tolerant Bird Percentage	Disease Mortality (μ_D_)		50% μ_N_ 110% F	50% μ_N_ 125% F	50% μ_N_ 150% F
Iiwi	High	0%	93%	0.03	0.3	0.4	0.5
		1%	47%	0.4	**1.1**	**1.3**	**1.4**
			23%	**1.1**	**1.6**	**1.6**	**1.7**
		5%	47%	0.7	**1.4**	**1.4**	**1.5**
			23%	**1.2**	**1.6**	**1.6**	**1.7**
	Mid	0%	93%	0.01	0.3	0.5	0.9
		1%	47%	**4.1**	**65**	**82**	**92**
			23%	**63**	**94**	**96**	**99**
		5%	47%	**15**	**80**	**87**	**92**
			23%	**68**	**94**	**96**	**99**
Amakihi	High	0%	68%	0.2	**2.3**	**2.7**	**3.2**
		1%	2.5%	**2.1**	**4.2**	**4.4**	**4.7**
		5%	2.5%	**2.7**	**4.3**	**4.5**	**4.7**
	Mid	0%	68%	0.1	**6.4**	**10**	**16**
		1%	2.5%	**15**	**24**	**25**	**26**
		5%	2.5%	**15**	**24**	**25**	**26**

μ_D_, the malaria-induced mortality from Samuel et al. 2015 [26] of 93% for non-tolerant Iiwi, and 68% for non-tolerant Amakhi; μ_N_, adult bird natural mortality; F, fecundity.

The combination of malaria tolerance with release of sterile or incompatible male mosquitoes produced complex patterns based on species, malaria induced mortalities, and elevation (Tables 5 and 6). Either malaria tolerance or sterile/incompatible male mosquitoes alone maintained or substantially increased Amakihi populations (PGR ≥ 1) at high and mid elevations and combining both actions did not produce adverse results, although there was no obvious synergy. The pattern was mixed for more disease-sensitive species, such as Iiwi. If the malaria fatality rate was reduced to less than 25%, Iiwi maintained stable populations in high elevation and recovered in mid elevation. The addition of sterile/incompatible male mosquitoes had insignificant or negative effects for Iiwi in both elevations, depending on the level of mating competition. However, if the malaria mortality was higher than 25%, incorporating sterile/incompatible mosquitoes benefited Iiwi populations, not by increasing malaria-tolerant birds, but by reducing wild mosquito abundance and helping malaria-susceptible birds. Due to the different level of selection pressure, highly competitive sterile/incompatible male mosquitoes assisted Iiwi populations in high elevation, but not Iiwi in mid-elevation forests (Tables 5 and 6).

## Discussion

Our previous research predicted that climate change will increase malaria transmission in mid-elevation forests causing additional impacts on native bird abundance [14]. Here, we extend these results by showing that climate change and resulting intensity of malaria transmission has a direct connection with the effectiveness of future malaria mitigation strategies. Among all of the single mitigation strategies, our simulations indicated that evolution of malaria tolerance, application of sterile/incompatible male mosquitoes, or use of refractory mosquitoes could be successful conservation actions for maintaining current levels of high-elevation native birds with moderate (Amakihi) or high malaria sensitivity (Iiwi) by 2100. We found that management actions such as predator removal or feral pig control may, temporarily or longer-term, postpone population declines until alternative strategies can be developed. In mid-elevation forests which are favorable for mosquito demographics and malaria parasite development, high rates of malaria infection reduced the effectiveness of many mitigation strategies. As a result, most single mitigation strategies will likely be unsuccessful in maintaining or increasing mid-elevation bird populations.

Our results indicated that development of malaria tolerance is one of the potential future scenarios for native Hawaiian species to survive future climate change. Currently, susceptibility to avian malaria varies among species of Hawaiian forest birds with mortality exceeding 90% in highly-susceptible Iiwi [6,26]. By contrast, Apapane and most Amakihi populations showed moderate mortality to avian malaria [6,26,36,62], likely explaining their higher abundance in mid-elevation forests where malaria transmission is higher. However, Amakihi at low elevations of Hawai’i have undergone a population explosion due to evolved malaria tolerance [21,24,26,28–30,63]. Interestingly, in this study we found that Apapane populations at mid and high elevation were not substantially impacted by future increases in avian malaria transmission (results not shown), suggesting that Apapane may have also developed (or perhaps always had) sufficient malaria tolerance to persist even under future disease increases. We found that Apapane and low-elevation Amakihi have sufficient ability to cope with malaria infection; therefore, no mitigation actions are needed to maintain these populations during this century.

If other species of Hawaiian birds were able to evolve malaria tolerance then future population declines and extinctions might be avoided. However, the potential for evolution of malaria-tolerance in many honeycreepers likely depends on complex factors including population demographics, selection pressure, genetic diversity, landscape level gene flow, habitat quality, food resources, predation, and competition [24,30,32]. Our results suggest assuming evolution of malaria tolerance in most Hawaiian species is likely overly optimistic for several reasons. First, our simulations did not consider any fitness costs for the development of malaria-tolerance [34,64]. Second, the reduction in malaria mortality that tolerant birds need to achieve is substantial for malaria sensitive Hawaiian populations like Iiwi. Limited improvement in the malaria fatality rate (μ_D_ = 70%) was not sufficient to reverse predicted population declines for high or mid elevations. So far, large reductions in malaria fatality rates have only been documented in low-elevation Amakihi, and the specific genotype(s) responsible for malaria tolerance has not been determined [30]. Based on the history of population extinction, declines, and restricted distribution for endemic Hawaiian birds caused by avian malaria it seems unlikely that many native species will be able to evolve malaria tolerance. Finally, species that develop tolerance would become a disease reservoir that can infect susceptible mosquitoes and transmit disease to the remaining susceptible birds [42].

Release of sterile/incompatible male mosquitoes provided another promising strategy to combat avian malaria transmission. Our simulations showed that sufficient release of sterile/incompatible male mosquitoes can result in dramatic declines in mosquito abundance in mid elevation during the initial part of the century (Fig 1), allowing populations of Amakihi and Iiwi to dramatically increase. However, success of this strategy depends heavily on mating competition of sterile/incompatible males compared to wild males and egg mortality rates, which are currently unknown for *Culex* mosquitoes. If the mating competition is low, it would require extended release periods and a large number of sterile/incompatible males to successfully reduce mosquito abundance and malaria transmission. In addition, the cost and feasibility of releasing sufficient mosquito numbers for a substantial portion of the year over a large heterogeneous, forested landscape and for long periods of time requires further research and cost benefit analysis. We suspect that development of aerial methods of mosquito release using manned or unmanned aircraft will be required to implement this strategy on the mountainous Hawaiian landscape. An important benefit of this strategy would be a dramatic reduction in the abundance of *Culex* mosquitoes; substantially reducing the potential future risk of newly introduced vector-borne diseases (e.g., West Nile Virus) to Hawaiian honeycreepers.

We found development of refractory *Culex* mosquitoes provided an alternative approach to protect Hawaiian forest birds. However, the long-term success of this action depended on whether the refractory mosquitoes could completely (in mid elevation) or largely (≥ 90% in high-elevation) replace the wild mosquito population. Although most research has focused on *Aedes* or *Anopheles* mosquitoes (which transmit dengue fever or human malaria) it has been reported that *Culex quinquefasciatus*, the avian malaria vector in Hawai’i, could also express the transposable element required for the gene drive mechanism [65]. The development of the CRISPAR Cas9 gene editing system as a gene drive for refractory genes is a potential game changer depending on the further development of system safeguards and regulatory acceptance [66–68]. However, implementing this strategy in *Culex* mosquitoes still requires additional technological advances including: 1) identification of refractory (anti-pathogen) gene(s) for avian malaria; 2) regulations and acceptance of CRISPR technology; and 3) conducting field tests to determine the efficiency of this method (no field tests have yet been conducted) [61,69]. In contrast to the release of sterile/incompatible males, this strategy is predicted to leave a high abundance of mosquitoes on the landscape. While these mosquitoes would be unable to transmit avian malaria they could be effective vectors of other diseases that could impact Hawaiian honeycreepers.

Introduced predators have played an important role in the decline of many insular bird populations [70]. In Hawai’i, introduced mammals such as black rats, feral cats, and mongooses prey on eggs, fledglings, or even adult birds [71]. One study of Oahu Elepaio (*Chasiempis ibidis*) found that rodent control improved female survival (nearly 50%), fecundity (> 100%), and nest success (near 100%) [71]. In contrast, no difference in nest success or abundance was detected in native forest birds during predator control at Hakalau Forest National Wildlife Refuge from 1996 to 1999 [71]. A rat control experiment using Kipukas (patches of native forest surrounded by lava flows) on Hawai’i Island during 2012 (Jessie Knowlton, Pers. Comm.) showed no significant improvement in nest success for Apapane, Amakihi, and Iiwi. Thus, potential impacts of predator removal on Hawaiian birds may be minor and/or depend on the specific ecological situation and predator characteristics [36,71]. We simulated a large range of potential demographic improvements for Amakihi and Iiwi by reducing adult natural (non-malaria) mortality and increasing fecundity. Whether such improvements can be achieved in practice is unknown, but previous studies suggest these goals are unlikely. Even with optimistic demographic improvements, our simulations showed predator removal alone was generally not sufficient to restore forest bird populations, especially for disease sensitive species. However, predator control was complementary to other mitigation actions, such as feral pig control (Table 1) or evolution of malaria tolerance (Table 7), provided bird populations have the genetic capability to develop tolerance. Currently, the costs and success of conducting long-term, landscape scale predator removal programs in Hawaiian forests are unknown.

Previous research [4–6,13] demonstrated that avian malaria is a key factor affecting the elevational distribution, dramatic decline, and extinction of many native Hawaiian forest birds. Development of malaria parasites and mosquito demography are closely linked to climate [11,27]; therefore, future climate changes are expected to exacerbate current malaria impacts on forest birds by extending season length and intensity of malaria transmission, as well as expanding the range of infection into high-elevation refugia. Benning et al. [22] first recognized the significance of malaria risks posed by future climate change in the Hawaiian system. They predicted a 2°C increase in temperature by 2100 would dramatically reduce the malaria-free zone found on several Hawaiian Islands, causing additional extinctions of many endemic species. Fortini et al. [25] used a temperature based species distribution model and the A1B climate scenario, with 2.6°C increase by 2100, to show that future habitat for many Hawaiian species will substantially decline as climate warms, purportedly as a result of increasing malaria risk. Despite the dire predictions of Benning et al. [22] and Fortini et al. [25], our analyses using a mechanistic mosquito-malaria-forest bird model for the Island of Hawai’i predicted higher future malaria transmission because temperatures used by Benning et al. [22] and Fortini et al. [25] are likely conservative (compared to a higher temperature RCP8.5 projection) and the impact of higher rainfall on mosquito dynamics for the A1B projection [14]. Despite these quantitative differences our results qualitatively agreed with previous studies [14,22,25]: many threatened or endangered Hawaiian forest birds currently found in high-elevation disease-free forests will likely be extirpated, or severely reduced in both mid- and high-elevation forests as global temperatures and avian malaria transmission increase during the 21^st^ century.

To counter these future threats to endemic Hawaiian forest birds requires the development and implementation of significant new conservation actions. Our results indicated the potential success of alternative strategies varied with the susceptibility of avian species to malaria, future climatic projections, and elevation. Apapane, which have both lower susceptibility to malaria and favorable population demographics compared to other species, were likely to persist at relatively high population levels during the 21^st^ century despite increased malaria risk caused by climate change [14]. Therefore, conservation programs aimed specifically at Apapane were not likely necessary. In contrast, Amakihi, with intermediate levels of malaria susceptibility and capacity for demographic response, may be a suitable species for evaluating conservation actions in mid-elevation forests where they are currently relatively abundant. In addition, low-elevation Amakihi are one of the few Hawaiian species that have evolved tolerance to malaria infection; providing future hope that other Hawaiian species may also adapt to malaria. Iiwi, which have limited demographic ability to compensate for their high malaria fatality rate (> 90%), are currently abundant only in high-elevation forests where malaria transmission is limited. Their high susceptibility means that future increases in malaria transmission will severely jeopardize this iconic species. Our results indicated conservation actions that maintain Iiwi populations in high-elevation forests under adverse climate projections (RCP8.5) would also benefit less susceptible species (Apapane and Amakihi). More importantly they would likely benefit other malaria-sensitive threatened and endangered Hawaiian honeycreepers that currently only exist in these disease refugia. In mid-elevation forests, single mitigation strategies were unsuccessful in maintaining (PGR near 1) or restoring (PGR >> 10) the scarce Iiwi populations. Instead integrated mitigation strategies were required to substantially recover (PGR >> 10) Iiwi to levels similar to those found in high-elevation forests.

### Conclusions

Our results suggested that long-term malaria mitigation should focus on maintaining malaria-sensitive Iiwi under the RCP8.5 climatic projection. This may be a worst case climate scenario and success in maintaining Iiwi population is also likely to maintain other populations of threatened and endangered Hawaiian honeycreepers, as well as less sensitive species such as Amakihi and Apapane. Second, our results indicated that mitigation strategies should especially focus on high-elevation forests as this is currently the only remaining habitat where abundant highly susceptible Iiwi and other critical populations of honeycreepers are found [1]. Fortunately, delays in climate warming suggest that malaria intensity at high-elevations will not substantially increase for a few decades [14]. As a result, we found that single mitigation strategies including evolution of malaria tolerance, mosquito reduction, or malaria transmission control by refractory mosquitoes were effective at high elevation. Third, different mitigation strategies may be needed for mid-elevation forests due to the differences in malaria infection risk, both currently and in the future. Implementation of single mitigation strategies at mid elevation including feral pig control or release of sterile/incompatible male mosquitoes provided a benefit to native bird populations for several decades, after which other conservation solutions or integrated mitigation strategies will be required. Fourth, mosquito intervention strategies produced different long-term outcomes in terms of mosquito abundance. While both strategies may be effective in reducing malaria infection in Hawaiian birds, using refractory mosquitoes results in much higher mosquito abundance than using sterile/incompatible males. Further consideration regarding the long-term risk for transmission of other vector-borne diseases is needed for these different outcomes. Fifth, the best long-term solution for native honeycreepers, without human intervention, is to evolve malaria-tolerance. Unfortunately, without better understanding of the genetic mechanism(s) that facilitate evolution of malaria tolerance it is currently impossible to determine whether this adaptation is feasible, which species might be able to develop tolerance, and how rapidly tolerance might evolve. Our results for Iiwi indicated that malaria mortality would need to decline dramatically, perhaps unrealistically so, for successful evolution by species with high malaria sensitivity. We also caution that adaptation for most of the threatened and endangered Hawaiian species may be unlikely given the small size of their remaining populations and the likelihood that evolved tolerance in more common species will accelerate the demise of species that are not able to adapt because the common species would serve as a disease reservoir. To date the historical evidence suggests that extinction from malaria is considerably more common in Hawai’i than adaptation. Thus, remaining native forest birds may benefit most if research, development, and assessment of alternative mitigation strategies is accelerated rather than delayed.

## Supporting Information

S1 Data Data sets for Figs 1, 2, 3, 4, S1 and S2. (XLSX)

S1 Fig The predicted Iiwi density (per km^2^) for feral pig control (green line) under alternative climatic projections (RCP8.5, A1B, RCP4.5) at high elevation. No pig control (red line) shows the predicted Iiwi abundance without pig management.
(TIF)

S2 Fig The predicted Amakihi density (per km^2^) for feral pig control (green line) under alternative climatic projections (RCP8.5, A1B, RCP4.5) at high elevation. No pig control (red line) shows the predicted Amakihi abundance without pig management.
(TIF)

S1 Table The population growth rate (PGR) of Iiwi for malaria tolerance in mid and high elevation based on future climatic projections (RCP8.5, A1B, RCP4.5), alternative reduction of malaria-induced mortality (μ_D_), and the initial frequency of tolerant birds in the population (1%, 5%, and 10%). (DOCX)

S2 Table The population growth rate (PGR) of Amakihi for malaria tolerance in mid and high elevation based on future climatic projections (RCP8.5, A1B, RCP4.5) and alternative initial frequency of tolerant birds in the population (1%, 5%, and 10%). (DOCX)

S3 Table The final percentage of the malaria-tolerant birds for different elevation, future climatic projections (RCP8.5, A1B, RCP4.5), malaria induced mortality (μ_D_), and initial percentage of tolerant birds in the population (1%, 5%, and 10%). (A) Iiwi. (B) Amakihi.
(DOCX)

S4 Table The population growth rate (PGR) of Iiwi and Amakihi from predator removal based on elevation, future climatic projections (RCP8.5, A1B, RCP4.5), and different reductions in natural mortality (75%, and 50% μ_N_) with increases in fecundity (105%, 110%, 125%, and 150% of current F). (DOCX)

S5 Table The population growth rate (PGR) for Iiwi and Amakihi for feral pig control based on elevation and future climatic projections (RCP8.5, A1B, RCP4.5). (DOCX)

S6 Table The population growth rate (PGR) for Iiwi and Amakihi for *Aedes japonicus japonicus* establishment based on elevation, future climatic projections (RCP8.5, A1B, RCP4.5), and different competitiveness (95% and 90%) between *Aedes* larvae and *Culex* larvae. (DOCX)

S7 Table The population growth rate (PGR) for Iiwi and Amakihi for sterile or incompatible male mosquito based on elevation, future climatic projections (RCP8.5, A1B, and RCP4.5), and combinations of release months, the number of male released per km^2^, mating competition between the sterile or incompatible male and wild male mosquitoes, and the egg mortality achieved by such mating. (DOCX)

S8 Table The population growth rate (PGR) for Iiwi and Amakihi for refractory mosquitoes based on elevation, future climatic projections (RCP8.5, A1B, RCP4.5), and the percentage of infected refractory mosquitoes (Ref% = 50%, 80%, 90%, 95% and 100%). (DOCX)
