# Supplementary figures and images for: Mitigating Future Avian Malaria Threats to Hawaiian Forest Birds from Climate Change
Source: PLoS One. 2017 Jan 6;12(1):e0168880. doi: 10.1371/journal.pone.0168880 (PMC5218566; doi:10.1371/journal.pone.0168880)

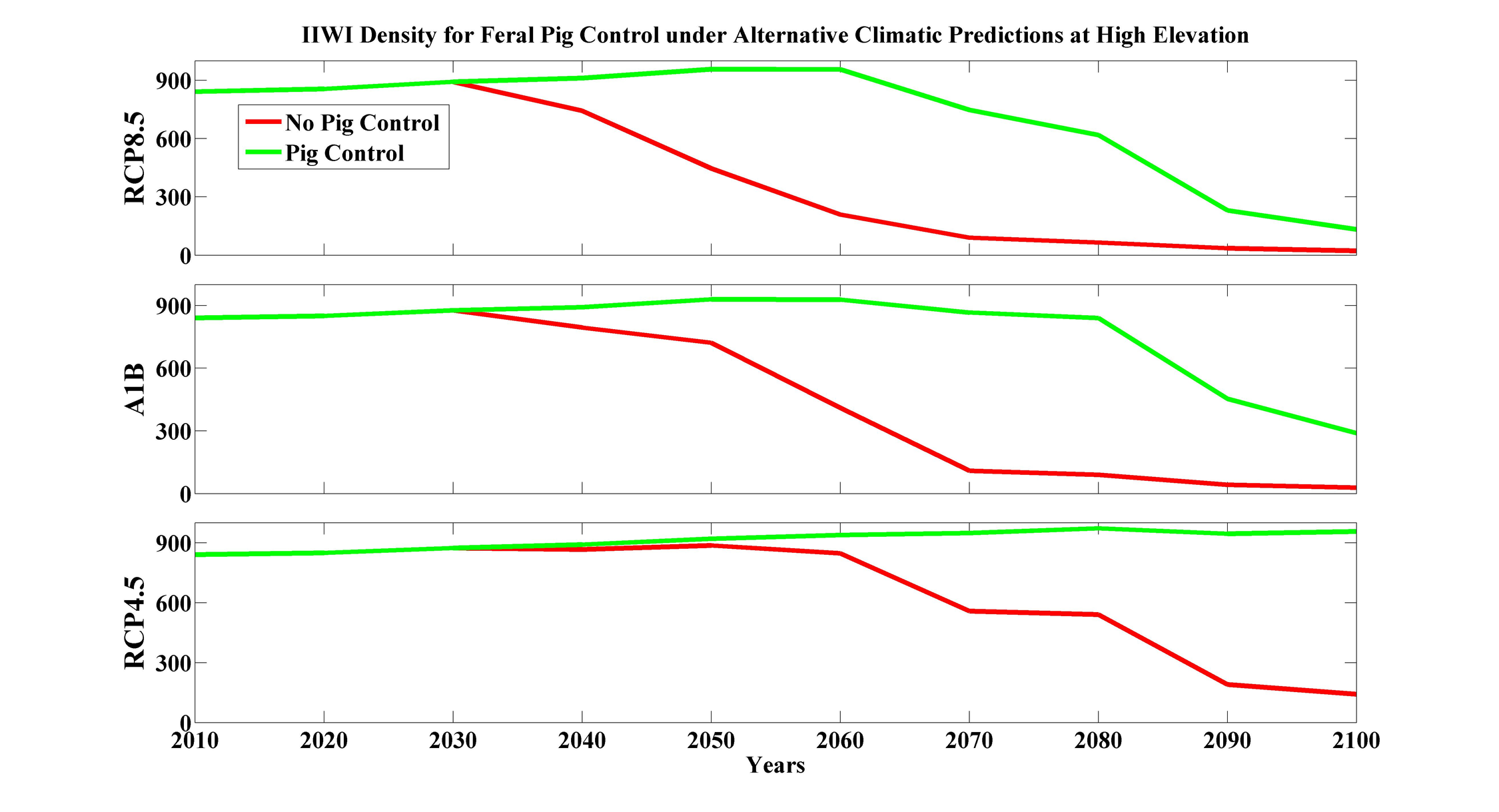

Supplement: S1 Fig — No pig control (red line) shows the predicted Iiwi abundance without pig management. (TIF) [file pone.0168880.s002.tif]

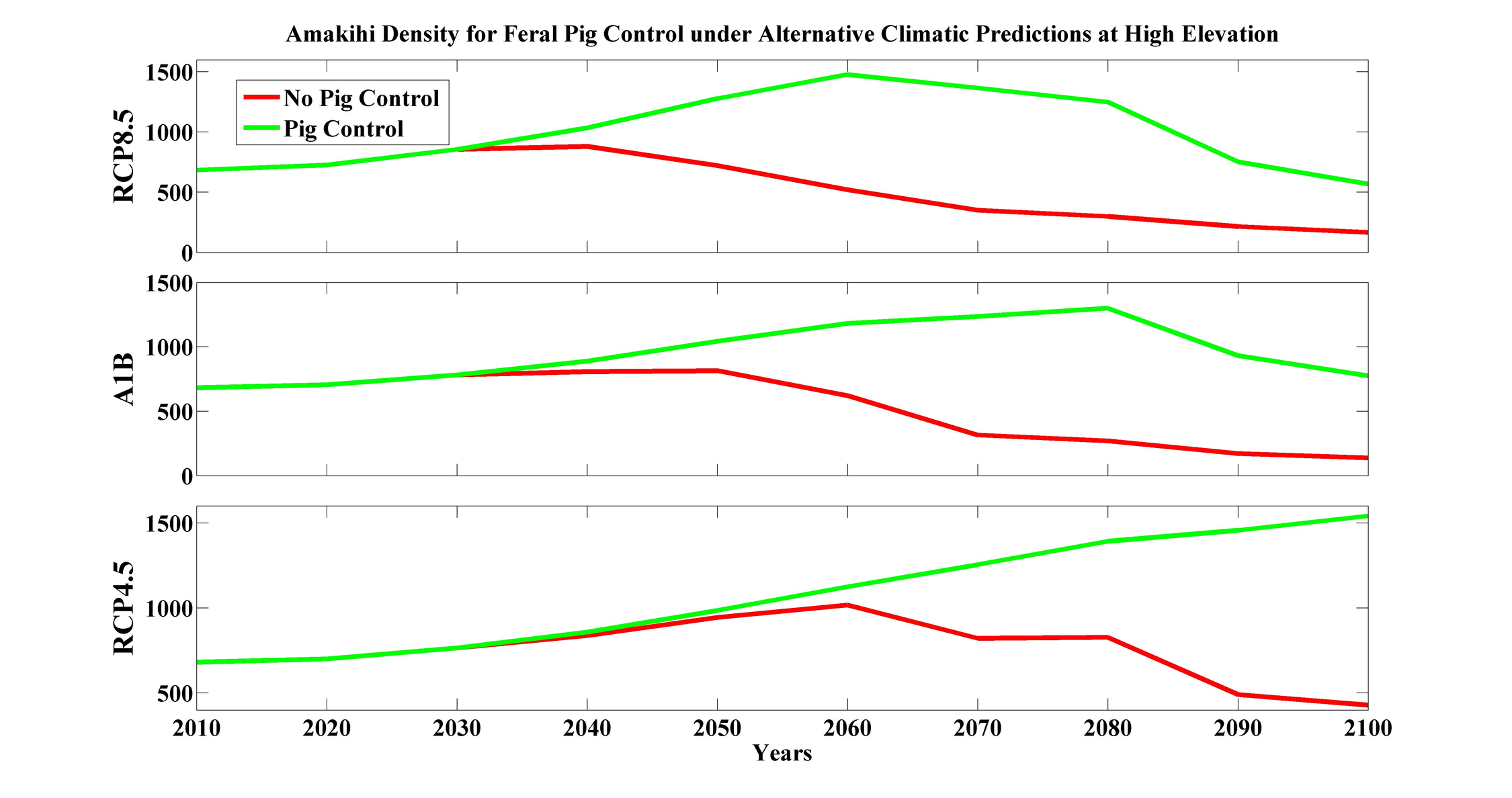

Supplement: S2 Fig — No pig control (red line) shows the predicted Amakihi abundance without pig management. (TIF) [file pone.0168880.s003.tif]
