# Supplementary material for: Mitigating Future Avian Malaria Threats to Hawaiian Forest Birds from Climate Change
Source: PLoS One. 2017 Jan 6;12(1):e0168880. doi: 10.1371/journal.pone.0168880 (PMC5218566; doi:10.1371/journal.pone.0168880)
Supplement: S1 Table — (DOCX) [file pone.0168880.s004.docx]

S1 Table. The population growth rate (PGR) of Iiwi for malaria tolerance in mid and high elevation based on future climatic projections (RCP8.5, A1B, RCP4.5), alternative reduction of malaria-induced mortality (μ_D_), and the initial frequency of tolerant birds in the population (1%, 5%, and 10%).

| Species | Elevation | Climate | μ_D_ = 93% | μ_D_ = 70% | | | μ_D_ = 47% | | | μ_D_ = 23% | | |
| --- | --- | --- | --- | --- | --- | --- | --- | --- | --- | --- | --- | --- |
|  |  |  |  | 1% | 5% | 10% | 1% | 5% | 10% | 1% | 5% | 10% |
| Iiwi | High | RCP8.5 | 0.03 | 0.04 | 0.1 | 0.2 | 0.4 | 0.7 | 0.8 | **1.1** | **1.2** | **1.2** |
|  |  | A1B | 0.03 | 0.1 | 0.1 | 0.1 | 0.2 | 0.5 | 0.6 | 0.8 | **1.1** | **1.1** |
|  |  | RCP4.5 | 0.2 | 0.2 | 0.2 | 0.3 | 0.2 | 0.4 | 0. 6 | 0.4 | 0.8 | 0.9 |
|  | Mid | RCP8.5 | 0.01 | 0.04 | 0.1 | 0.3 | **4.1** | **15** | **23** | **63** | **68** | **68** |
|  |  | A1B | 0.01 | 0.04 | 0.2 | 0.3 | **4.3** | **16** | **24** | **63** | **67** | **68** |
|  |  | RCP4.5 | 0.01 | 0.04 | 0.2 | 0.3 | **4.4** | **16** | **24** | **63** | **67** | **68** |

μ_D_, the malaria-induced mortality; 1%, 5%, 10% – the initial tolerant bird proportion
