# Supplementary material for: Mitigating Future Avian Malaria Threats to Hawaiian Forest Birds from Climate Change
Source: PLoS One. 2017 Jan 6;12(1):e0168880. doi: 10.1371/journal.pone.0168880 (PMC5218566; doi:10.1371/journal.pone.0168880)
Supplement: S2 Table — (DOCX) [file pone.0168880.s005.docx]

S2 Table. The population growth rate (PGR) of Amakihi for malaria tolerance in mid and high elevation based on future climatic projections (RCP8.5, A1B, RCP4.5) and alternative initial frequency of tolerant birds in the population (1%, 5%, and 10%).

| Species | Elevation | Climate | Model Baseline | Initial Tolerant Bird Proportion | | |
| --- | --- | --- | --- | --- | --- | --- |
|  |  |  |  | 1% | 5% | 10% |
| Amakihi | High | RCP8.5 | 0.2 | **2.1** | **2.7** | **2.8** |
|  |  | A1B | 0.2 | **1.1** | **2.0** | **2.4** |
|  |  | RCP4.5 | 0.6 | 0.8 | **1.3** | **1.7** |
|  | Mid | RCP8.5 | 0.1 | **15** | **15** | **15** |
|  |  | A1B | 0.1 | **15** | **15** | **15** |
|  |  | RCP4.5 | 0.1 | **15** | **15** | **15** |

Estimated malaria mortality of 68% and 2.5% for malaria-susceptible and malaria-tolerant respectively (Samuel et al. 2015); Model baseline, all malaria-susceptible birds, having 68% disease mortality (μ_D_)
