# Supplementary material for: Mitigating Future Avian Malaria Threats to Hawaiian Forest Birds from Climate Change
Source: PLoS One. 2017 Jan 6;12(1):e0168880. doi: 10.1371/journal.pone.0168880 (PMC5218566; doi:10.1371/journal.pone.0168880)
Supplement: S3 Table — (A) Iiwi. (B) Amakihi. (DOCX) [file pone.0168880.s006.docx]

S3 Table. The final percentage of the malaria-tolerant birds for different elevation, future climatic projections (RCP8.5, A1B, RCP4.5), malaria induced mortality (μ_D_), and initial percentage of tolerant birds in the population (1%, 5%, and 10%).

1. Iiwi

| Species | Elevation | Climate | μ_D_ = 93% | μ_D_ = 70% | | | μ_D_ = 47% | | | μ_D_ = 23% | | |
| --- | --- | --- | --- | --- | --- | --- | --- | --- | --- | --- | --- | --- |
|  |  |  |  | 1% | 5% | 10% | 1% | 5% | 10% | 1% | 5% | 10% |
| Iiwi | High | RCP8.5 | 0 | 44 | 79 | 88 | 94 | 98 | 99 | 99 | 99 | 99 |
|  |  | A1B | 0 | 29 | 66 | 80 | 85 | 95 | 97 | 97 | 99 | 99 |
|  |  | RCP4.5 | 0 | 9.2 | 36 | 54 | 32 | 72 | 84 | 63 | 89 | 93 |
|  | Mid | RCP8.5 | 0 | 71 | 93 | 96 | 100 | 100 | 100 | 100 | 100 | 100 |
|  |  | A1B | 0 | 71 | 93 | 96 | 100 | 100 | 100 | 100 | 100 | 100 |
|  |  | RCP4.5 | 0 | 71 | 93 | 96 | 100 | 100 | 100 | 100 | 100 | 100 |

μ_D_, the malaria-induced mortality; 1%, 5%, 10%, the initial tolerant bird proportion

1. Amakihi

| Species | Elevation | Climate | Model Baseline | Initial Tolerant Bird Proportion | | |
| --- | --- | --- | --- | --- | --- | --- |
|  |  |  |  | 1% | 5% | 10% |
| Amakihi | High | RCP8.5 | 0 | 94 | 98 | 99 |
|  |  | A1B | 0 | 86 | 96 | 97 |
|  |  | RCP4.5 | 0 | 28 | 68 | 81 |
|  | Mid | RCP8.5 | 0 | 100 | 100 | 100 |
|  |  | A1B | 0 | 100 | 100 | 100 |
|  |  | RCP4.5 | 0 | 100 | 100 | 100 |

Estimated malaria mortality of 68% and 2.5% for malaria-susceptible and malaria-tolerant respectively (Samuel et al. 2015); Model baseline, all malaria-susceptible birds, having 68% disease mort
