# Supplementary material for: Mitigating Future Avian Malaria Threats to Hawaiian Forest Birds from Climate Change
Source: PLoS One. 2017 Jan 6;12(1):e0168880. doi: 10.1371/journal.pone.0168880 (PMC5218566; doi:10.1371/journal.pone.0168880)
Supplement: S4 Table — (DOCX) [file pone.0168880.s007.docx]

S4 Table. The population growth rate (PGR) of Iiwi and Amakihi from predator removal based on elevation, future climatic projections (RCP8.5, A1B, RCP4.5), and different reductions in natural mortality (75%, and 50% μ_N_) with increases in fecundity (105%, 110%, 125%, and 150% of current F))

| Species | Elevation | Climate | Model Baseline | 75% * μ_N_ | | | | 50% * μ_N_ | | | |
| --- | --- | --- | --- | --- | --- | --- | --- | --- | --- | --- | --- |
|  |  |  |  | 105% * F | 110% * F | 125% * F | 150% * F | 105% * F | 110% * F | 125% * F | 150% *F |
| Iiwi | High | RCP8.5 | 0.03 | 0.1 | 0.1 | 0.2 | 0.3 | 0.3 | 0.3 | 0.4 | 0.5 |
|  |  | A1B | 0.03 | 0.1 | 0.1 | 0.2 | 0.3 | 0.3 | 0.3 | 0.4 | 0.5 |
|  |  | RCP4.5 | 0.2 | 0.3 | 0.4 | 0.5 | 0.7 | 0.5 | 0.6 | 0.7 | 0.9 |
|  | Mid | RCP8.5 | 0.01 | 0.1 | 0.1 | 0.1 | 0.2 | 0.3 | 0.3 | 0.5 | 0.9 |
|  |  | A1B | 0.01 | 0.1 | 0.1 | 0.1 | 0.2 | 0.3 | 0.3 | 0.5 | 0.9 |
|  |  | RCP4.5 | 0.01 | 0.1 | 0.1 | 0.1 | 0.2 | 0.3 | 0.4 | 0.5 | 1.0 |
| Amakihi | High | RCP8.5 | 0.2 | **1.0** | **1.2** | **1.6** | **2.3** | **2.2** | **2.3** | **2.7** | **3.2** |
|  |  | A1B | 0.2 | 0.9 | **1.1** | **1.5** | **2.2** | **2.0** | **2.2** | **2.6** | **3.1** |
|  |  | RCP4.5 | 0.6 | **1.6** | **1.7** | **2.2** | **2.8** | **2.5** | **2.6** | **3.0** | **3.5** |
|  | Mid | RCP8.5 | 0.05 | 0.7 | **1.0** | **2.5** | **7.6** | **5.1** | **6.4** | **10** | **16** |
|  |  | A1B | 0.05 | 0.7 | **1.0** | **2.5** | **7.7** | **5.2** | **6.4** | **10** | **16** |
|  |  | RCP4.5 | 0.05 | 0.8 | **1.1** | **2.7** | **7.9** | **5.4** | **6.6** | **11** | **16** |

μ_N_, adult bird natural mortality; F, fecundity; Model baseline, without predator removal: 100%* μ_N_ and 100%*F
