# Supplementary material for: Mitigating Future Avian Malaria Threats to Hawaiian Forest Birds from Climate Change
Source: PLoS One. 2017 Jan 6;12(1):e0168880. doi: 10.1371/journal.pone.0168880 (PMC5218566; doi:10.1371/journal.pone.0168880)
Supplement: S5 Table — (DOCX) [file pone.0168880.s008.docx]

S5 Table. The population growth rate (PGR) for Iiwi and Amakihi for feral pig control based on elevation and future climatic projections (RCP8.5, A1B, RCP4.5).

| Species | Elevation | Climate | No Pig Control | Pig Control |
| --- | --- | --- | --- | --- |
| Iiwi | High | RCP8.5 | 0.03 | 0.2 |
|  |  | A1B | 0.03 | 0.3 |
|  |  | RCP4.5 | 0.2 | **1.1** |
|  | Mid | RCP8.5 | 0.01 | 0.01 |
|  |  | A1B | 0.01 | 0.01 |
|  |  | RCP4.5 | 0.01 | 0.01 |
| Amakihi | High | RCP8.5 | 0.2 | 0.8 |
|  |  | A1B | 0.2 | **1.1** |
|  |  | RCP4.5 | 0.6 | **2.3** |
|  | Mid | RCP8.5 | 0.1 | 0.1 |
|  |  | A1B | 0.1 | 0.1 |
|  |  | RCP4.5 | 0.1 | 0.1 |
