# Supplementary material for: Mitigating Future Avian Malaria Threats to Hawaiian Forest Birds from Climate Change
Source: PLoS One. 2017 Jan 6;12(1):e0168880. doi: 10.1371/journal.pone.0168880 (PMC5218566; doi:10.1371/journal.pone.0168880)
Supplement: S6 Table — (DOCX) [file pone.0168880.s009.docx]

S6 Table. The population growth rate (PGR) for Iiwi and Amakihi for *Aedes japonicus* *japonicus* establishment based on elevation, future climatic projections (RCP8.5, A1B, RCP4.5), and different competitiveness (95% and 90%) between *Aedes* larvae and *Culex* larvae.

| Species | Elevation | Climate | Model Baseline  (100% * K_L_ ) | 5% Reduction  (95% * K_L_) | 10% Reduction  (90% * K_L_) |
| --- | --- | --- | --- | --- | --- |
| Iiwi | High | RCP8.5 | 0.03 | 0.03 | 0.03 |
|  |  | A1B | 0.03 | 0.04 | 0.04 |
|  |  | RCP4.5 | 0.2 | 0.2 | 0.2 |
|  | Mid | RCP8.5 | 0.01 | 0.01 | 0.01 |
|  |  | A1B | 0.01 | 0.01 | 0.01 |
|  |  | RCP4.5 | 0.01 | 0.01 | 0.01 |
| Amakihi | High | RCP8.5 | 0.2 | 0.3 | 0.3 |
|  |  | A1B | 0.2 | 0.2 | 0.2 |
|  |  | RCP4.5 | 0.6 | 0.7 | 0.7 |
|  | Mid | RCP8.5 | 0.1 | 0.1 | 0.1 |
|  |  | A1B | 0.1 | 0.1 | 0.1 |
|  |  | RCP4.5 | 0.1 | 0.1 | 0.1 |

K_L_, elevation-specific mosquito larvae carrying capacity
