# Supplementary material for: Mitigating Future Avian Malaria Threats to Hawaiian Forest Birds from Climate Change
Source: PLoS One. 2017 Jan 6;12(1):e0168880. doi: 10.1371/journal.pone.0168880 (PMC5218566; doi:10.1371/journal.pone.0168880)
Supplement: S7 Table — (DOCX) [file pone.0168880.s010.docx]

S7 Table. The population growth rate (PGR) for Iiwi and Amakihi for sterile or incompatible male mosquito based on elevation, future climatic projections (RCP8.5, A1B, and RCP4.5), and combinations of release months, the number of male released per km^2^, mating competition between the sterile or incompatible male and wild male mosquitoes, and the egg mortality achieved by such mating.

| Species | Elevation | Climate | Months of Release | Number of Mosquitoes  (km^2^) | Competition  Coefficient | Egg Mortality | PGR |
| --- | --- | --- | --- | --- | --- | --- | --- |
| Iiwi | High | RCP8.5 | Baseline | | | | 0.03 |
|  |  |  | 12 | 500 | 1.0 | 0.95 | 0.8 |
|  |  |  | 12 | 500 | 1.0 | 0.99 | **1.0** |
|  |  |  | 12 | 750 | 0.5 | 0.95 | 0.6 |
|  |  |  | 12 | 750 | 0.5 | 0.99 | **1.0** |
|  |  | A1B | Baseline | | | | 0.03 |
|  |  |  | 12 | 500 | 0.8 | 0.95 | 0.7 |
|  |  |  | 12 | 500 | 0.8 | 0.99 | **1.1** |
|  |  |  | 12 | 750 | 0.5 | 0.95 | 0.4 |
|  |  |  | 12 | 750 | 0.5 | 0.99 | **1.2** |
|  |  | RCP4.5 | Baseline | | | | 0.2 |
|  |  |  | 12 | 500 | 0.5 | 0.90 | **1.2** |
|  |  |  | 9 | 750 | 0.5 | 0.90 | **1.2** |
|  | Mid | RCP8.5 | Baseline | | | | 0.01 |
|  |  |  | 9 | 500 | 0.5 | 0.95 | **7.6** |
|  |  | A1B | Baseline | | | | 0.05 |
|  |  |  | 9 | 500 | 0.5 | 0.95 | **6.3** |
|  |  | RCP4.5 | Baseline | | | | 0.05 |
|  |  |  | 9 | 500 | 0.5 | 0.95 | **78** |
| Amakihi | High | RCP8.5 | Baseline | | | | 0.3 |
|  |  |  | 12 | 500 | 0.5 | 0.90 | **1.0** |
|  |  |  | 12 | 500 | 0.5 | 0.95 | **1.4** |
|  |  | A1B | Baseline | | | | 0.2 |
|  |  |  | 12 | 500 | 0.5 | 0.90 | 0.8 |
|  |  |  | 12 | 500 | 0.5 | 0.95 | **1.1** |
|  |  |  | 12 | 750 | 0.5 | 0.90 | **1.1** |
|  |  | RCP4.5 | Baseline | | | | 0.6 |
|  |  |  | 12 | 500 | 0.5 | 0.90 | **2.5** |
|  |  |  | 9 | 750 | 0.5 | 0.90 | **2.4** |
|  | Mid | RCP8.5 | Baseline | | | | 0.05 |
|  |  |  | 9 | 500 | 0.5 | 0.90 | **1.3** |
|  |  | A1B | Baseline | | | | 0.05 |
|  |  |  | 9 | 500 | 0.5 | 0.90 | **1.1** |
|  |  | RCP4.5 | Baseline | | | | 0.05 |
|  |  |  | 9 | 500 | 0.5 | 0.90 | **1.7** |
