# Supplementary material for: Mitigating Future Avian Malaria Threats to Hawaiian Forest Birds from Climate Change
Source: PLoS One. 2017 Jan 6;12(1):e0168880. doi: 10.1371/journal.pone.0168880 (PMC5218566; doi:10.1371/journal.pone.0168880)
Supplement: S8 Table — (DOCX) [file pone.0168880.s011.docx]

S8 Table. The population growth rate (PGR) for Iiwi and Amakihi for refractory mosquitoes based on elevation, future climatic projections (RCP8.5, A1B, RCP4.5), and the percentage of infected refractory mosquitoes (Ref% = 50%, 80%, 90%, 95% and 100%)

| Species | Elevation | Climate | Model Baseline | Ref = 50% | Ref = 80% | Ref = 90% | Ref = 95% | Ref = 100% |
| --- | --- | --- | --- | --- | --- | --- | --- | --- |
| Iiwi | High | RCP8.5 | 0.03 | 0.06 | 0.2 | **1.3** | **1.3** | **1.4** |
|  |  | A1B | 0.03 | 0.09 | 0.95 | **1.3** | **1.3** | **1.3** |
|  |  | RCP4.5 | 0.2 | 0.9 | **1.3** | **1.3** | **1.3** | **1.3** |
|  | Mid | RCP8.5 | 0.01 | 0.01 | 0.01 | 0.02 | 0.05 | **78** |
|  |  | A1B | 0.01 | 0.01 | 0.01 | 0.02 | 0.1 | **78** |
|  |  | RCP4.5 | 0.01 | 0.01 | 0.02 | 0.04 | 0.8 | **78** |
| Amakihi | High | RCP8.5 | 0.2 | 0.4 | **1.1** | **2.8** | **2.9** | **3.0** |
|  |  | A1B | 0.2 | 0.5 | **2.1** | **2.8** | **2.8** | **2.8** |
|  |  | RCP4.5 | 0.6 | **1.8** | **2.6** | **2.6** | **2.6** | **2.6** |
|  | Mid | RCP8.5 | 0.1 | 0.1 | 0.1 | 0.1 | 0.3 | **16** |
|  |  | A1B | 0.1 | 0.1 | 0.1 | 0.2 | 0.6 | **16** |
|  |  | RCP4.5 | 0.1 | 0.1 | 0.1 | 0.3 | **1.7** | **16** |

Ref%, the infected refractory mosquito percentage; Model Baseline, no refractory mosquitoes
